# Interplay between collective behavior and spreading dynamics on complex networks

**DOI:** 10.1063/1.4766677

**Published:** 2012-11-26

**Authors:** Kezan Li, Zhongjun Ma, Zhen Jia, Michael Small, Xinchu Fu

**Affiliations:** 1 School of Mathematics and Computing Science, Guilin University of Electronic Technology, Guilin 541004, People's Republic of China; 2 College of Science, Guilin University of Technology, Guilin 541004, People's Republic of China; 3 School of Mathematics and Statistics, The University of Western Australia, Crawley, WA 6009, Australia; 4 Department of Mathematics, Shanghai University, Shanghai 200444, People's Republic of China

## Abstract

There are certain correlations between collective behavior and spreading dynamics on some real complex networks. Based on the dynamical characteristics and traditional physical models, we construct several new bidirectional network models of spreading phenomena. By theoretical and numerical analysis of these models, we find that the collective behavior can inhibit spreading behavior, but, conversely, this spreading behavior can accelerate collective behavior. The spread threshold of spreading network is obtained by using the Lyapunov function method. The results show that an effective spreading control method is to enhance the individual awareness to collective behavior. Many real-world complex networks can be thought of in terms of both collective behavior and spreading dynamics and therefore to better understand and control such complex networks systems, our work may provide a basic framework.

From the real world, we can find many examples in which collective behavior and spreading behavior appear simultaneously and interplay with one another. In the stock market, the prices of different stocks will increase or decrease synchronously if relevant political canard spreads. Conversely, the price fluctuation also influences the spreading of relevant political canard. With the fast spread of an infectious disease in society, the times of avoiding assemblage and washing hand, etc., of people will increase in a synchronous way to protect themselves. On the other hand, this synchronous response will weaken the disease spread to some degree. In order to precisely control these collective and spreading behaviors and understand their interplay from the viewpoint of mathematics, the first step should be the construction of a suitable model which can display similar properties for these real dynamical networks. So, in this work, we will provide some mathematical models and address correlation between the collective and spreading dynamics on complex networks. The research results show that our models correspond closely with many real dynamical complex networks, and an effective spreading control method is to enhance the individual awareness to collective behavior.

## I. INTRODUCTION

It is well known that many real biological and social systems can be considered as dynamical complex networks. For examples, the daily activities of cows (eating, lying down, and standing) can be modeled by a dynamical network, where local dynamics of each cow is described by an oscillator of a piecewise linear dynamical system.^1^ The authors in Ref. 1 not only studied interesting dynamics such as synchronization but also developed some biological predictions. Many other examples can be found in Ref. 2. Now, let us ask: when an infectious disease spreads among these cows, what is the impact on their synchronous behavior? Can then this synchronous behavior weaken or strengthen the disease spread process? We feel that these problems can be resolved by examining the interplay between the collective and spreading dynamics on complex networks.

It is an interesting and important topic to consider the interplay between different dynamical behaviors appearing in complex networks. The correlation between traffic flow and epidemic spreading on complex networks was investigated numerically and theoretically in Ref. 3, for the case where the epidemic incidence was shaped by traffic-flow conditions and epidemic pathways were defined and driven by flows. The results in Ref. 3 provided a general framework for us to understand the spreading processes on complex traffic networks. We have investigated mathematically the correlation between the dynamical synchronization and the epidemic behavior on complex networks,^4^ and a very explicit condition for synchronization with respect to the epidemic rate was obtained. However, in this case, we only considered a special collective behavior, i.e., global synchronization. Moreover, the correlation between the dynamical synchronization and the epidemic behavior is unidirectional, i.e., the spreading behavior can influence the dynamical synchronization, but not vice versa. So, in this paper, we will extend our former research work to consider that the correlation between the collective behavior and the spread behavior is bidirectional and address further phase synchronization which may be a more general collective behavior.

In this work, we first propose a general bidirectional model of collective behavior and spreading dynamics on complex networks in Sec. II. Then in Secs. III–V, two concrete models are provided and studied, respectively, corresponding to two collective behaviors of dynamical behavior network, i.e., global synchronization and phase synchronization. By using the Lyapunov function method, we investigate the spread threshold of spreading on a network. In Sec. VI, we investigate finally the control problem of spreading behavior and provide an effective control strategy.

## II. A GENERAL BIDIRECTIONAL MODEL

A general coupled model of collective and spreading behaviors on complex networks can be described as

$$\begin{array}{c} \dot{X}(t)=F(X(t),c(t)),\\ \dot{Y}(t)=G(Y(t),E(t)),\\ \dot{c}(t)=H(Y(t),E(t)), \end{array}$$ (1)

where $X(t)=(x_{1}(t),x_{2}(t),\ldots ,x_{N}(t))$ with $x_{i}(t)\in {\mathbb{R}}^{n}$ denotes the state variable of the *i*-th individual in a dynamical behavior network with size *N*, which can exhibit collective behavior under suitable conditions, which is a necessary condition in this work. The coupling strength c(t)>0. The mapping $F:({\mathbb{R}}^{\mathrm{Nn}},\mathbb{R})\to {\mathbb{R}}^{\mathrm{Nn}}$ controls the dynamical change process of state variable *X*(*t*). In the second equality of Eq. (1), $Y(t)\in {\mathbb{R}}^{d}$ denotes the density variable of a spreading process on a network with maximal degree *d*. The variable E(t)∈ℝ is the error of collective behavior among state variables $x_{i}(t),i=1,2,\ldots ,N$ and may be defined in different forms. The mapping $G:({\mathbb{R}}^{d},\mathbb{R})\to {\mathbb{R}}^{d}$ characterizes the dynamical change process of density variable *Y*(*t*). For the last equality, the function $H:({\mathbb{R}}^{d},\mathbb{R})\to \mathbb{R}$ defines an adaptive law of the coupling strength *c*(*t*).

**FIG. 1.**
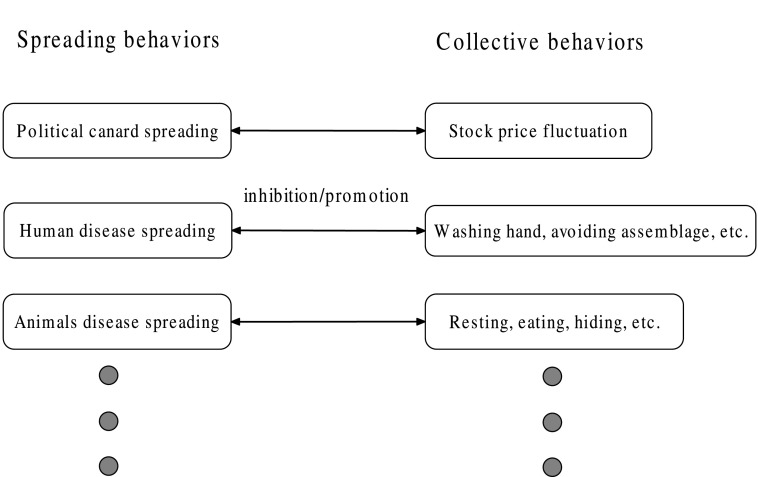
Relationship between some groups of collective and spreading behaviors. For each group, bidirectional arrowhead implies interactional relationship. Many other probable cases are not listed and this is denoted by the ellipsis.

System (1) gives a bidirectional model between collective behavior and spread dynamics, where the dynamical behavior process *X*(*t*) can play a role in spread behavior *Y*(*t*) by embedding the error *E*(*t*), and the spread behavior *Y*(*t*) influences the dynamical behavior process *X*(*t*) by changing its coupling strength *c*(*t*). In real life, many dynamical phenomena can be described and explained by system (1). For example, when a political canard spreads, many relevant stocks will increase or decrease their prices synchronously for the explicit benefit of their corresponding corporations by closer communication. Conversely, the collective price fluctuation also accelerates or decelerates the spreading of relevant political canards. Similarly, when a certain infectious disease breaks out, people or animals will take some collective protective measures such as washing hands frequently, avoiding assemblage, resting frequently, etc. At the same time, these collective behaviors will further influence the disease spread. Fig. 1 gives a schematic diagram of three groups of collective and spreading behaviors which are interrelated closely, where the arrowhead shows the reliant relation between them.

In Secs. III–V, we will consider two important collective behaviors, i.e., global synchronization and phase synchronization, and investigate the interplay between them and corresponding spreading behaviors. Undoubtedly, to deal with these problems the first step is that system (1) should be described precisely with the corresponding mappings *F, G, H,* and *E*.

## III. GLOBAL SYNCHRONIZATION AND SPREADING DYNAMICS

Before constructing a concrete system, we should make the following basic assumptions. There is a weakly linear coupling between individuals in the dynamical behavior network in the beginning stage when spreading begins. And, there exists an interactional relationship between a dynamical behavior network and a spreading network, i.e., inhibiting or promoting each other.

Based on these assumptions, the model of SIS spread synchronization proposed in Ref. 4 and the framework of general model (1), we can construct a concrete system as

$$\{\begin{array}{cc} {\dot{x}}_{i}(t)=f(x_{i}(t))+c(t)\sum_{j=1}^{N}a_{\mathrm{ij}}Hx_{j}(t), & \\ {\dot{I}}_{k}(t)=\lambda \phi (t)k[1-I_{k}(t)]\Theta (t)-I_{k}(t), & \\ \dot{c}(t)=\beta I(t)E_{1}(t), & \end{array}$$ (2)

where *i* = 1, 2,…, *N*, *k* = 1, 2,…,*d*. Compared to the general model (1), we have $X(t)=(x_{1}(t),x_{2}(t),\ldots ,x_{N}(t))$ and $Y(t)={\left(I_{1}(t),I_{2}(t),\ldots ,I_{d}(t)\right)}^{T}$, correspondingly. Moreover, $x_{i}(t)\in {\mathbb{R}}^{n}$ denotes the state variable of the *i*-th node at the time *t*, and the function f(·) defines the local dynamics of each node and is supposed to be chaotic. The function c(t)>0 is the coupling strength and the matrix $H\in {\mathbb{R}}^{n\times n}$ represents the inner-coupling matrix which is a constant 0 – 1 matrix linking coupled variables, and we assume it is positive. The coupling matrix $A={\left(a_{\mathrm{ij}}\right)}_{N\times N}$ with zero-sum rows shows the coupling configuration of the network. If nodes *i* and *j* are connected, then $a_{\mathrm{ij}}=a_{\mathrm{ji}}=1$; otherwise $a_{\mathrm{ij}}=a_{\mathrm{ji}}=0$. The diagonal elements of the coupling matrix *A* are

$$a_{\mathrm{ii}}=-\sum_{j=1,j\neq i}^{N}a_{\mathrm{ij}}=-k_{i},\;\;\;i=1,2,\ldots ,N,$$ (3)

where $k_{i}$ denotes the degree of node *i*. With these assumptions, the eigenvalues^5^ of matrix *A* can be given by $0=\lambda_{1}\geq \lambda_{2}\geq \cdots \lambda_{N}$. The global synchronization error is set as

$$E(t)=\frac{1}{N}\sum_{j=1}^{N}\frac{∥s(t)-x_{j}(t){∥}^{2}}{1+∥s(t)-x_{j}(t){∥}^{2}}\in [0,1),$$ (4)

where *s*(*t*) is the synchronous state of the dynamical behavior network. Then, we define ϕ(t)=(1−α)E(t)+α with constant α∈(0,1), and $E_{1}(t)=\sum_{j=1}^{N}∥s(t)-x_{j}(t){∥}^{2}$ in the third equation of system (2).

The variables $I_{k}(t)$ denote the density of infected nodes (individuals) with connectivity (contact) *k* at time *t* and $I(t)=\sum_{k=1}^{d}p(k)I_{k}(t)$ is the total infectious density. The spread rate λ∈(0,1] denotes the probability with which each susceptible node is infected if it is connected to one infected node. The term Θ(t) gives the probability that a randomly chosen link emanating from a node leads to an infected node. Moreover, Θ(t) has the form

$$\Theta (t)=\sum_{k^{\prime }=1}^{d}\frac{k^{\prime }p(k^{\prime })I_{k^{\prime }}(t)}{〈k〉},$$ (5)

where the average degree $〈k〉=\sum_{k=1}^{d}kp(k)$. By this form, we mean that the connectivities of nodes in the spreading network are uncorrelated. The parameter β>0.

The initial condition of system (2) can be set as follows. The initial state $x_{i}(0)$ is chosen randomly from the real numbers and $I_{k}(0)=\rho_{k},\;c(0)=\sigma$ with $0<\rho_{k}\ll 1$ and 0<σ≪1.

The physical meaning of model (2) was explained in detail in our former paper.^4^ Besides, the additional term ϕ(t)=(1−α)E(t)+α in the second equation of model (2) denotes the admission rate,^6^ as the information of synchronization can be considered as a kind of individual awareness (or the risk perception). When all individuals achieve synchronization, i.e., E(t)→0 as t→∞, then the admission rate achieves the minimum α. Smaller value of parameter α means greater awareness to collective behavior. The case α=1 shows there is no awareness to the information of synchronization.

The infection control behavior of individuals within the community can be quantified by the variable ϕ(t)—this is the degree to which the behavior of the individual acts to reduce their risk of infection from others (and also risk of infecting others for a disease with a latent period): the rate of infection λ becomes ϕ(t)λ. Nonetheless, this individual behavior is a manifestation of the individuals behavior (through, for example, wearing of face masks, modification of hygiene practice, sharing of utensils, and washing of hands), and this is something which can be observed by others. There is a collectivization in ones response—if more people are wearing face masks in public it becomes more acceptable to do so and one is more likely to follow suit: or vice versa. Hence, the dynamical behavior of this parameter ϕ(t) exhibits a synchronization which, in term, influences the disease dynamics.

Now, we will address two basic properties of system (2), i.e., spread threshold of the spreading network and synchronization stability of the dynamical behavior network. By using a similar analytical method presented in Ref. 7, we can obtain easily that the spread threshold is

$$\lambda_{c}=\frac{〈k〉}{\alpha 〈k^{2}〉}>\frac{〈k〉}{〈k^{2}〉}.$$ (6)

In order to prove this spread threshold from the view of mathematics we should adopt a global stability analysis method, which will be shown in the next section. For the dynamical behavior network, if $\lambda >\lambda_{c}$, then all $x_{i}(t),i=1,2,\ldots ,N$ will realize synchronization globally and asymptotically, as we will investigate in Sec. IV.

We now give some numerical examples to investigate model (2). Without loss of generality, we assume that the local dynamics of the dynamical behavior network are identical and defined as the chaotic Lorenz oscillation.^8^ From the point of view of a physical spread transmission process, it is rather difficult to justify this assumption. But we just use it for numerical simulations. This oscillation can be presented as

$$\{\begin{array}{cc} \frac{dx_{1}(t)}{\mathrm{dt}}=a_{1}(x_{2}(t)-x_{1}(t)), & \\ \frac{dx_{2}(t)}{\mathrm{dt}}=a_{2}x_{1}(t)-x_{2}(t)-x_{1}(t)x_{3}(t), & \\ \frac{dx_{3}(t)}{\mathrm{dt}}=x_{1}(t)x_{2}(t)-a_{3}x_{3}(t), & \end{array}$$ (7)

where parameters $a_{1}=10,a_{2}=28,a_{3}=(8/3)$. And the inner-coupling matrix *H* is chosen as the identity matrix.

**FIG. 2.**
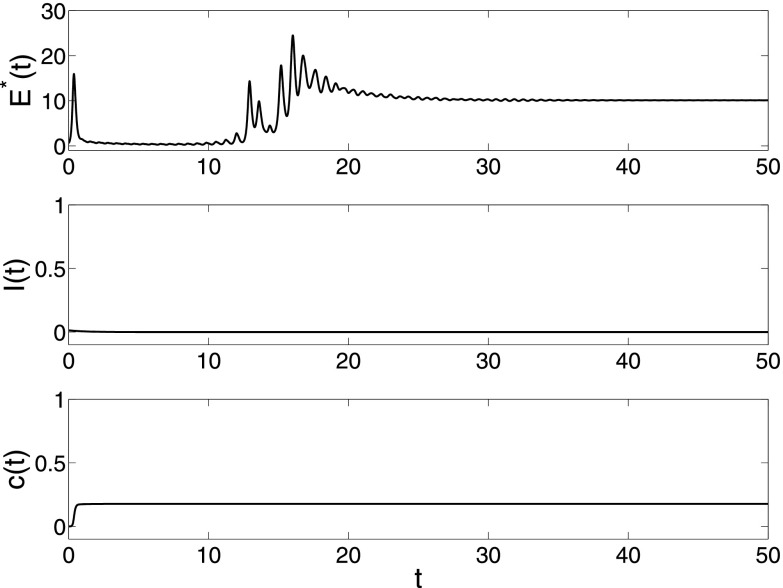
The changes of synchronization error $E^{*}(t)=\frac{1}{N-1}\sum_{i=2}^{N}∥x_{1}(t)-x_{i}(t)∥$, epidemic prevalence *I*(*t*) and coupling strength *c*(*t*) in model (2) under parameters N=200,λ=0.05,α=0.5,β=0.001,σ=0.01. Both the spreading network and dynamical behavior network have the same BA network structure with minimal degree 3. There are only infected nodes with degree 3 and infection density $\rho_{3}=0.03$, and the other nodes are all susceptible in the beginning stage.

**FIG. 3.**
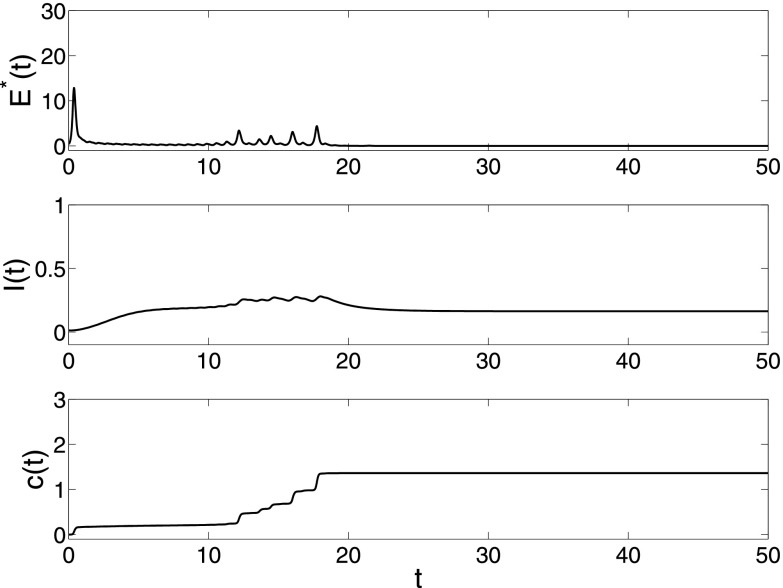
The changes of $E^{*}(t)$, *I*(*t*) and *c*(*t*) in model (2) under parameters N=200,λ=0.3,α=0.5,β=0.001,σ=0.01.

First, we consider that the spreading network and dynamical behavior network having the same network topological structure. This implies that the individuals in the spreading network and dynamical behavior network are identical. For example, when human disease spreads, the reactive collective behaviors of washing hand, avoiding assemblage, etc., are also generated by people within the community. The network structure embedded in model (2) is set to be the BA network^9^ with size *N* = 200. This network evolves from initial network with size $m_{0}=4$ and we add each new node with *m* = 3 new edges. Other parameters are chosen as $\rho_{3}=0.03,\rho_{k}=0,k\neq 3$ and σ=0.01,β=0.001. Fig. 2 gives a simulation under λ=0.05,α=0.5, where the spreading does not become endemic and synchronization is not realized. In this case, the individuals do not exhibit collective behavior since the spread will die out and its prevalence is too small to keep them close. By increasing the spreading rate to λ=0.3 in Fig. 3, the synchronization is achieved and spreading also becomes endemic. Moreover, a very interesting phenomenon is that the total density *I*(*t*) is oscillating, which is also found in adaptive epidemic networks.^10,11^ If the collective behavior fails to influence the spreading, i.e., setting α=1, then the total density *I*(*t*) will converge monotonically to a larger value. From this simulation, we can see that the collective behavior can inhibit greatly the spreading behavior.

**FIG. 4.**
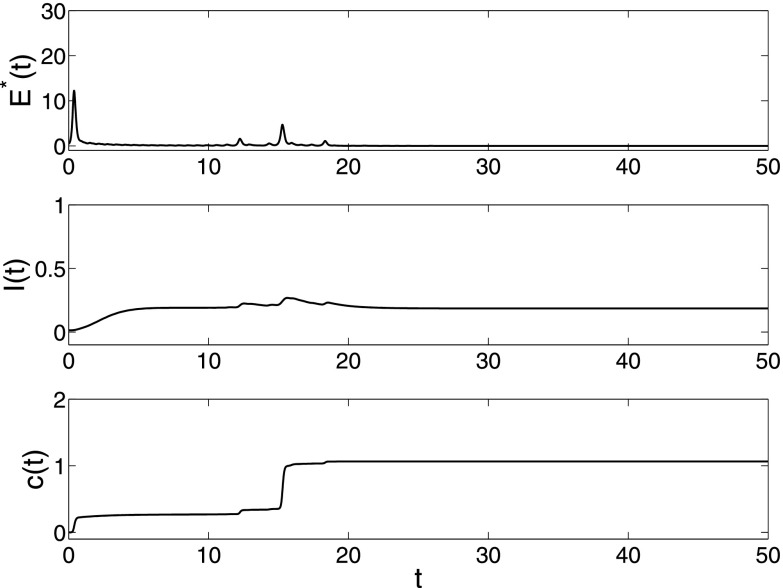
The changes of synchronization error $E^{*}(t)=\frac{1}{N_{2}-1}\sum_{i=2}^{N_{2}}∥x_{1}(t)-x_{i}(t)∥$, epidemic prevalence *I*(*t*) and coupling strength *c*(*t*) in model (2) under parameters λ=0.3,α=0.5,β=0.001,σ=0.01. Both the spreading network and dynamical behavior network have different BA network structures with sizes $N_{1}=200,N_{2}=300$, respectively.

Next, we study an expert case in which the spreading network and dynamical behavior network have different network topological structures. This means that the individuals in the spreading network and the dynamical behavior network are nonidentical. For example, in the event of a political canard, the spreading network consists of individual people, while the stocks can be regarded as the corresponding dynamical behavior network of the stock market. Let $N_{1},N_{2}$ denote the sizes of spreading network and dynamical behavior network both with BA mechanisms, respectively. With other parameters fixed, from Fig. 4 we can not find essential difference by only changing the network structure. The impact of the change of network structure on the spreading dynamics will be further discussed in detail in Sec. VI.

## IV. STABILITY OF GLOBAL SYNCHRONIZATION AND SPREADING DYNAMICS

In this section, we will study the global stability of equilibriums of model (2) by utilizing the method of global Lyapunov functions. Based on this analysis, we can get the spread threshold (6) for the spreading network and a synchronization condition for the dynamical behavior network.

For the dynamical behavior network in model (2), we first make the following preparations.

Suppose that $P=diag(p_{1},p_{2},\ldots ,p_{n})$ is a positive matrix. If there is a constant ξ, such that for all $x(t),y(t)\in {\mathbb{R}}^{n},\;t>0$, then, we always have that

$${\left(x-y\right)}^{T}P[f(x)-f(y)]\leq \xi {\left(x-y\right)}^{T}(x-y).$$

By letting $\bar{F}(t)={\left(f{\left(x_{1}(t)\right)}^{T}-f{\left(s(t)\right)}^{T},\ldots ,f{\left(x_{1}(t)\right)}^{T}-f{\left(s(t)\right)}^{T}\right)}^{T},\;G(t)={\left(g^{T},\ldots ,g^{T}\right)}^{T}$, and $e(t)={\left(e_{1}^{T},\ldots ,e_{N}^{T}\right)}^{T}$, then the error system of dynamical behavior network in model (2) can be written as

$$\dot{e}(t)=\bar{F}(t)+c(t)(A⊗H)e(t)+(I_{N}⊗I_{n})G(t),$$ (8)

where ⊗ is Kronecker product and $I_{N}$ denotes *N*-order identity matrix.

For the spreading network in model (2), we set $\beta_{\mathrm{kj}}=\frac{\lambda kjp(j)}{〈k〉},i,j=1,2,\ldots ,d$, and nonnegative matrices

$$M(I)=\phi (t){\left(\beta_{\mathrm{kj}}(1-I_{k})\right)}_{d\times d}$$ (9)

and

$$M_{0}={\left(\beta_{\mathrm{kj}}\right)}_{d\times d}.$$ (10)

By setting $I=(I_{1},I_{2},\ldots ,I_{d})\in {\mathbb{R}}^{d}$, then the spreading network in model (2) can be rewritten as in a more compact form

$$\dot{I}(t)=M(I)I-I.$$ (11)

By simple computation, we know that the nonnegative matrix $M_{0}$ has eigenvalues as $\mu_{1}=\mu_{2}=\cdots =\mu_{d-1}=0$ and $\mu_{d}=\lambda \frac{\Sigma_{i=1}^{d}i^{2}p(i)}{〈k〉}=\lambda \frac{〈k^{2}〉}{〈k〉}$. Define

$$R_{0}=\phi (t)\rho (M_{0}),$$ (12)

where ρ denotes the spectral radius. Then we have $R_{0}=\lambda \phi (t)\frac{〈k^{2}〉}{〈k〉}$, which is the basic reproduction number for this spreading network that will be shown later. When the synchronization of the dynamical behavior network achieves stability, $R_{0}$ will converges to fixed value $\lambda \alpha \frac{〈k^{2}〉}{〈k〉}$, from which we can get the spread threshold (6).

Obviously, the matrix $M(I_{0})$ is irreducible, where $I_{0}=(0,0,\ldots ,0)\in {\mathbb{R}}^{d}$. Define

$$\Gamma =\{(I_{1},I_{2},\ldots ,I_{d})\in {\mathbb{R}}^{d}|0\leq I_{k}\leq 1,k=1,2,\ldots ,d\},$$

and let $\Gamma^{o}$ denote the interior of Γ. The spreading network in model (2) is said to be uniformly persistent^12^ in $\Gamma^{o}$, if there exists a constant γ∈(0,1) such that $\underset{t\to \infty }{\mathrm{liminf}}I_{k}(t)>\gamma$ for all *k* provided $(I_{1}(0),I_{2}(0),\ldots ,I_{d}(0))\in \Gamma^{o}$. Since the spreading network can be reduced to a particular case of multi-group epidemic model, we have the following stability analysis mainly enlightened by the work in Ref. 12, which have solved the uniqueness and global stability of a multi-group SIR epidemic model.

**Theorem 1.** If $R_{0}\leq 1$, then $I_{0}$ is the unique equilibrium of the spreading network in model (2) and it is globally stable in Γ. If $R_{0}>1$, then $I_{0}$ is unstable and this network is uniformly persistent in $\Gamma^{o}$.

*Proof*. By noting that

$$\begin{array}{c} \rho (M(I))=\phi (t)\rho \{{\left(\beta_{\mathrm{kj}}(1-I_{k})\right)}_{d\times d}\}\\ <\phi (t)\rho (M_{0}), \end{array}$$

if $R_{0}=\phi (t)\rho (M_{0})\leq 1$, then the equation *M*(*I*) *I* = *I* has only the zero solution $I=I_{0}$.

Let $\omega =(\omega_{1},\omega_{2},\ldots ,\omega_{d})$ be a left eigenvector of $M_{0}$ corresponding to $\rho (M_{0})$, i.e.,

$$\omega \rho (M_{0})=\omega M_{0}.$$ (13)

Since $M_{0}$ is irreducible, then $\omega_{i}>0$ for *i* = 1, 2,…, *d*. Define the following function:

$$V(t)=\sum_{k=1}^{d}\omega_{k}I_{k}.$$ (14)

The derivative of *V*(*t*) with respect to *t* along the solution of the system (11) is given by

$$\begin{array}{c} \frac{dV(t)}{\mathrm{dt}}=\omega [M(I)I-I]\\ \leq \omega (\phi (t)M_{0}I-I)\\ =\phi (t)\omega M_{0}I-\omega I\\ =\phi (t)\rho (M_{0})\omega I-\omega I\\ =[\phi (t)\rho (M_{0})-1]\omega I\\ =(R_{0}-1)\omega I. \end{array}$$ (15)

If $R_{0}<1$, then $\frac{dV(t)}{\mathrm{dt}}=0$ means $I=I_{0}$. If $R_{0}=1$, then $\frac{dV(t)}{\mathrm{dt}}=0$ implies ωM(I)I=ωI. Assuming that $I\neq I_{0}$, then we can get that $\omega M(I)I<\omega \phi (t)M_{0}I=\omega I$. So, in this situation, the equation ωM(I)I=ωI is satisfied if and only if $I=I_{0}$. Therefore, when $R_{0}\leq 1$ the only compact invariant subset of the set $\left\{I|\frac{dV(t)}{\mathrm{dt}}=0\right\}$ is the singleton $\left\{I_{0}\right\}$. By LaSalle's Invariance Principle, $I_{0}$ is globally asymptotically stable.

If $R_{0}>1$ and $I\neq I_{0}$, by using Eq. (15) we have

$$\omega (\phi (t)M_{0}I-I)>0.$$

With the limitation $\lim_{I\to I_{0}}\omega [M(I)I-I]=\omega (\phi (t)M_{0}I-I)$, we can conclude that $\frac{dV(t)}{\mathrm{dt}}>0$ in a neighborhood of $I_{0}$ in $\Gamma^{o}$. So, in this case the equilibrium $I_{0}$ is unstable. By a similar discussion in Ref. 12, this instability means that this network is uniformly persistent in $\Gamma^{o}$.

**Theorem 2.** If $R_{0}>1$, then there exists a unique endemic equilibrium $I^{*}$ of the spreading network in model (2), and it is globally asymptotically stable in $\Gamma^{o}$. Moreover, the synchronization manifold of the dynamical behavior network in this model is also globally asymptotically stable.

*Proof*. Since $S_{k}(t)+I_{k}(t)=1$ for *k* = 1, 2,…, *d*, the spreading network can be rewritten as

$${\dot{S}}_{k}(t)=1-S_{k}-\sum_{j}\phi (t)\beta_{\mathrm{kj}}S_{k}I_{j},$$ (16a)

$${\dot{I}}_{k}(t)=-I_{k}(t)+\sum_{j}\phi (t)\beta_{\mathrm{kj}}S_{k}I_{j}.$$ (16b)

And the error system of the dynamical behavior network is described by

$$\dot{e}(t)=\bar{F}(t)+c(t)(A⊗H)e(t)+(I_{N}⊗I_{n})G(t).$$ (17)

Let $(I_{1}^{*},I_{2}^{*},\ldots ,I_{d}^{*})\in \Gamma^{o}$ be an endemic equilibrium of the spreading network (16), and set ${\bar{\beta }}_{\mathrm{ij}}=\beta_{\mathrm{ij}}S_{i}^{*}I_{j}^{*}$, where $S_{i}^{*}(t)=1-I_{i}^{*}(t)$, then define a matrix

$$\bar{B}=\left(\begin{array}{cccc} \sum_{j\neq 1}{\bar{\beta }}_{1j} & -{\bar{\beta }}_{21} & \cdots & -{\bar{\beta }}_{d1}\\ -{\bar{\beta }}_{12} & \sum_{j\neq 2}{\bar{\beta }}_{2j} & \cdots & -{\bar{\beta }}_{d2}\\ \vdots & \vdots & \ddots & \vdots \\ -{\bar{\beta }}_{1d} & -{\bar{\beta }}_{2d} & \cdots & \sum_{j\neq d}{\bar{\beta }}_{\mathrm{dj}} \end{array}\right),$$ (18)

whose each column sum equals zero.

Construct a function as $V(t)=V_{1}(t)+V_{2}(t)$, with

$$V_{1}(t)=\sum_{k=1}^{d}v_{k}(S_{k}-S_{k}^{*}\;\ln\;S_{k}+I_{k}-I_{k}^{*}\;\ln\;I_{k}),$$ (19)

where $v_{k}>0$ is the cofactor of the *k*-th diagonal entry of $\bar{B}$ satisfying $\bar{B}{\left(v_{1},v_{2},\ldots ,v_{d}\right)}^{T}≜\bar{B}v=0$,^12^ and

$$V_{2}(t)=\frac{1}{2}e^{T}(t)(I_{N}⊗P)e(t)+\frac{1}{2}\bar{\beta }{\left(c_{0}-c(t)\right)}^{2},$$ (20)

where $\bar{\beta }=-\lambda_{2}\lambda_{\min}(PH)>0,\;\lambda_{\min}(PH)$ denotes the minimal eigenvalue of matrix *PH* and $c_{0}$ is a undetermined constant.

For $V_{1}(t)$, its derivative with respect to *t* along the solution of the system (16) is given by

$$\begin{array}{c} \frac{dV_{1}(t)}{\mathrm{dt}}=\sum_{k=1}^{d}v_{k}(1-S_{k}-\sum_{j}\phi (t)\beta_{\mathrm{kj}}S_{k}I_{j}-\frac{S_{k}^{*}}{S_{k}}(1-S_{k}-\sum_{j}\phi (t)\beta_{\mathrm{kj}}S_{k}I_{j})\\ -I_{k}+\sum_{j}\phi (t)\beta_{\mathrm{kj}}S_{k}I_{j}-\sum_{j}\phi (t)\beta_{\mathrm{kj}}S_{k}I_{j}\frac{I_{k}^{*}}{I_{k}}+I_{k}^{*})\\ =\sum_{k=1}^{d}v_{k}(S_{k}^{*}+\alpha \sum_{j}\beta_{\mathrm{kj}}S_{k}^{*}I_{j}^{*}-S_{k}-\sum_{j}\phi (t)\beta_{\mathrm{kj}}S_{k}I_{j}-\frac{S_{k}^{*}}{S_{k}}(S_{k}^{*}+\alpha \sum_{j}\beta_{\mathrm{kj}}S_{k}^{*}I_{j}^{*})\\ +S_{k}^{*}+\sum_{j}\phi (t)\beta_{\mathrm{kj}}S_{k}^{*}I_{j}+\sum_{j}\phi (t)\beta_{\mathrm{kj}}S_{k}I_{j}-I_{k}-\sum_{j}\phi (t)\beta_{\mathrm{kj}}S_{k}I_{j}\frac{I_{k}^{*}}{I_{k}}+\alpha \sum_{j}\beta_{\mathrm{kj}}S_{k}^{*}I_{j}^{*})\\ =\sum_{k=1}^{d}v_{k}(-S_{k}^{*}(\frac{S_{k}}{S_{k}^{*}}+\frac{S_{k}^{*}}{S_{k}}-2)+2\alpha \sum_{j}\beta_{\mathrm{kj}}S_{k}^{*}I_{j}^{*}-\sum_{j}\phi (t)\beta_{\mathrm{kj}}S_{k}I_{j}-\alpha \sum_{j}\beta_{\mathrm{kj}}\frac{{\left(S_{k}^{*}\right)}^{2}}{S_{k}}I_{j}^{*}\\ +\sum_{j}\phi (t)\beta_{\mathrm{kj}}S_{k}I_{j}-\sum_{j}\phi (t)\beta_{\mathrm{kj}}S_{k}I_{j}\frac{I_{k}^{*}}{I_{k}}+\sum_{j}\phi (t)\beta_{\mathrm{kj}}S_{k}^{*}I_{j}-I_{k}). \end{array}$$ (21)

Now, we will show that

$$\sum_{k=1}^{d}v_{k}(\sum_{j=1}^{d}\phi (t)\beta_{\mathrm{kj}}S_{k}^{*}I_{j}-I_{k})\leq \frac{E_{1}(t)}{N}\sum_{k,j=1}^{d}v_{k}\beta_{\mathrm{kj}}S_{k}^{*}I_{j}.$$ (22)

For the left-hand side of above inequality, we have

$$\begin{array}{c} \sum_{k=1}^{d}v_{k}(\sum_{j=1}^{d}\phi (t)\beta_{\mathrm{kj}}S_{k}^{*}I_{j}-I_{k})=\sum_{k=1}^{d}(\sum_{j=1}^{d}\phi (t)\beta_{\mathrm{jk}}S_{j}^{*}v_{j}-v_{k})I_{k}\\ \leq E(t)\sum_{k,j=1}^{d}v_{j}\beta_{\mathrm{jk}}S_{j}^{*}I_{k}+\sum_{k=1}^{d}(\alpha \sum_{j=1}^{d}\beta_{\mathrm{jk}}S_{j}^{*}v_{j}-v_{k})I_{k}\\ \leq \frac{E_{1}(t)}{N}\sum_{k,j=1}^{d}v_{k}\beta_{\mathrm{kj}}S_{k}^{*}I_{j}+\sum_{k=1}^{d}(\alpha \sum_{j=1}^{d}\beta_{\mathrm{jk}}S_{j}^{*}v_{j}-v_{k})I_{k}. \end{array}$$

To prove the inequality (22), it suffices to show

$$\alpha \sum_{j=1}^{d}\beta_{\mathrm{jk}}S_{j}^{*}v_{j}-v_{k}=0,\;k=1,2,\ldots ,d.$$

To this end, by using $\bar{B}v=0$ we consider

$$\begin{array}{c} \alpha \sum_{j=1}^{d}\beta_{\mathrm{jk}}S_{j}^{*}I_{k}^{*}v_{j}=\alpha \sum_{j\neq k}^{d}{\bar{\beta }}_{\mathrm{jk}}v_{j}+\alpha {\bar{\beta }}_{\mathrm{kk}}v_{k}\\ =\alpha \sum_{j\neq k}^{d}{\bar{\beta }}_{\mathrm{kj}}v_{k}+\alpha {\bar{\beta }}_{\mathrm{kk}}v_{k}\\ =\alpha \sum_{j=1}^{d}{\bar{\beta }}_{\mathrm{kj}}v_{k}\\ =\alpha \sum_{j=1}^{d}\beta_{\mathrm{kj}}S_{j}^{*}I_{k}^{*}v_{k}\\ =I_{k}^{*}v_{k}, \end{array}$$

that implies $\alpha \sum_{j=1}^{d}\beta_{\mathrm{jk}}S_{j}^{*}v_{j}-v_{k}=0$ for all *k*. Since

$$\frac{S_{k}}{S_{k}^{*}}+\frac{S_{k}^{*}}{S_{k}}-2\geq 0,$$

we get

$$-S_{k}^{*}(\frac{S_{k}}{S_{k}^{*}}+\frac{S_{k}^{*}}{S_{k}}-2)\leq 0,$$ (23)

and the equal sign holds if and only if $S_{k}=S_{k}^{*}$.

Using Eqs. (21)–(23) and noting that ϕ(t)≥α>0 and ${\bar{\beta }}_{\mathrm{ij}}=\beta_{\mathrm{ij}}S_{i}^{*}I_{j}^{*}$, we further obtain

$$\begin{array}{c} \frac{dV_{1}(t)}{\mathrm{dt}}\leq \frac{E_{1}(t)}{N}\sum_{k,j=1}^{d}v_{k}\beta_{\mathrm{kj}}S_{k}^{*}I_{j}+\alpha \sum_{k=1}^{d}v_{k}(2\sum_{j=1}^{d}{\bar{\beta }}_{\mathrm{kj}}-\sum_{j=1}^{d}{\bar{\beta }}_{\mathrm{kj}}\frac{S_{k}^{*}}{S_{k}}-\sum_{j=1}^{d}{\bar{\beta }}_{\mathrm{kj}}\frac{I_{j}S_{k}I_{k}^{*}}{I_{k}S_{k}^{*}I_{j}^{*}})\\ =\frac{\sum_{k,j=1}^{d}v_{k}\beta_{\mathrm{kj}}S_{k}^{*}I_{j}}{N}\sum_{i=1}^{N}e_{i}{\left(t\right)}^{T}e_{i}(t)+\alpha \sum_{k,j=1}^{d}v_{k}{\bar{\beta }}_{\mathrm{kj}}(2-\frac{S_{k}^{*}}{S_{k}}-\frac{I_{j}S_{k}I_{k}^{*}}{I_{k}S_{k}^{*}I_{j}^{*}}). \end{array}$$ (24)

Based on graph theory, the authors in Ref. 12 have proven that $\sum_{k,j=1}^{d}v_{k}{\bar{\beta }}_{\mathrm{kj}}(2-\frac{S_{k}^{*}}{S_{k}}-\frac{I_{j}S_{k}I_{k}^{*}}{I_{k}S_{k}^{*}I_{j}^{*}})\leq 0$ for positive $\beta_{\mathrm{ij}}$. And the above equal sign holds if and only if $S_{k}=S_{k}^{*},I_{k}=I_{k}^{*}$.

Now, let us turn to the derivative of $V_{2}(t)$ with respect to *t* along the solution of the system (16). By utilizing the similar analysis process present in Ref. 4, we can get

$$\frac{dV_{2}(t)}{\mathrm{dt}}\leq [\xi +c_{0}\lambda_{2}\lambda_{\min}(PH)\beta I(t)]\sum_{i=1}^{N}e_{i}{\left(t\right)}^{T}e_{i}(t).$$

From Theorem 1, we know that if $R_{0}>1$, then the spreading network in model (2) is uniformly persistent in $\Gamma^{o}$. Combining the continuity and this uniformly persistent property of function *I*(*t*), we can conclude that if $R_{0}>1$, then there is a constant $\bar{\gamma }>0$ such that $I(t)=\sum_{k=1}^{d}p(k)I_{k}(t)>\bar{\gamma }$ for all t>0. So, we can further obtain

$$\frac{dV_{2}(t)}{\mathrm{dt}}\leq [\xi +c_{0}\lambda_{2}\lambda_{\min}(PH)\beta \bar{\gamma }]\sum_{i=1}^{N}e_{i}{\left(t\right)}^{T}e_{i}(t).$$ (25)

Integrating the above discussions, we have

$$\begin{array}{c} \frac{dV(t)}{\mathrm{dt}}\leq [\xi +\frac{\sum_{k,j=1}^{d}v_{k}\beta_{\mathrm{kj}}S_{k}^{*}I_{j}}{N}+c_{0}\lambda_{2}\lambda_{\min}(PH)\beta \bar{\gamma }]\\ \times \sum_{i=1}^{N}e_{i}{\left(t\right)}^{T}e_{i}(t). \end{array}$$ (26)

Thus, we can select an adequately large constant $c_{0}$ such that $\frac{dV(t)}{\mathrm{dt}}\leq 0$. Moreover, form inequality (24) and (26), we know the largest invariant subset of

$$\left\{(S_{1},\ldots ,S_{d},I_{1},\ldots ,I_{d},e_{1},\ldots ,e_{N},c)|\frac{dV(t)}{\mathrm{dt}}=0\right\}$$ (27)

is the singleton $\left(S_{1}^{*},\ldots ,S_{d}^{*},I_{1}^{*},\ldots ,I_{d}^{*},0,\ldots ,0,c_{0}\right)$. By LaSalle's Invariance Principle, this equilibrium is globally asymptotically stable. So, the unique endemic equilibrium $I^{*}$ of the spreading network in model (2) is globally asymptotically stable in $\Gamma^{o}$, and the synchronization manifold of the dynamical behavior network is also globally asymptotically stable.

In addition, note here that we have obtained the basic reproduction number by a global analysis to SIS model on complex network, while the results of the literature^12^ are applicable to multi-group SIR model. This means that we have extended the analysis in Ref. 12 to a more general model.

## V. PHASE SYNCHRONIZATION AND SPREADING DYNAMICS

Compared to global synchronization discussed in Sec. IV, phase synchronization may be a more general collective behavior and more commonly observed in the real world. So, in this section, we will address this collective behavior and its influence on spreading behavior. Based on the famous Kuramoto model^13–15^ and the framework of general model (1), we can construct a concrete system as

$$\{\begin{array}{cc} {\dot{\theta }}_{i}(t)=\omega_{i}+c(t)\sum_{j=1}^{N}a_{\mathrm{ij}}\text{sin}(\theta_{j}(t)-\theta_{i}(t)), & \\ {\dot{I}}_{k}(t)=\lambda [(1-\alpha )(1-E(t))+\alpha ]k[1-I_{k}(t)]\Theta (t) & \\ \;\;\;\;\;\;\;\;\;\;-I_{k}(t), & \\ \dot{c}(t)=\beta I(t)(1-E(t)), & \end{array}$$ (28)

where *i* = 1, 2,…, *N*, *k* = 1, 2,…, *d*. The phase of the *i*-th individual is denoted by $\theta_{i}$, and $\omega_{i}$ represents its intrinsic frequency. Compared to the general model (1), we get $X(t)=(\theta_{1}(t),\theta_{2}(t),\ldots ,\theta_{N}(t))$ and $Y(t)={\left(I_{1}(t),I_{2}(t),\ldots ,I_{d}(t)\right)}^{T}$, correspondingly. The phase synchronization error is set as

$$E(t)=|\frac{1}{N}\sum_{j=1}^{N}e^{i\theta_{j}}|\in [0,1],$$ (29)

and the meaning of other mathematical symbols in model (28) is the same as that stated in Sec. III. If E(t)→1 as t→∞, then the dynamical behavior network achieves global phase synchronization. If E(t)→0, then the phases of all individuals are different from each other and no synchronization phenomenon exists in this dynamical behavior network. When E(t)→ε and 0<ε<1, this means that cluster synchronization appears with a proportion ε. Then, the spread threshold of the spreading network is

$$\lambda_{c}=\frac{1}{(1-\alpha )(1-\varepsilon )+\alpha }\times \frac{〈k〉}{〈k^{2}〉}>\frac{〈k〉}{〈k^{2}〉}.$$ (30)

The initial condition of system (28) can be set as follows. Without loss of generality, we choose the WS small-world network^16^ with probability *p* = 0.1 for rewiring links as the topology structure for the spreading network and dynamical behavior network. The initial phase $\theta_{i}(0)$ and intrinsic frequency $\omega_{i}$ are chosen uniformly from intervals (−π,π) and (−1/2, −1/2), respectively. The parameters α=0.8,β=1 and initial coupling strength *c*(0) = 0.1.

**FIG. 5.**
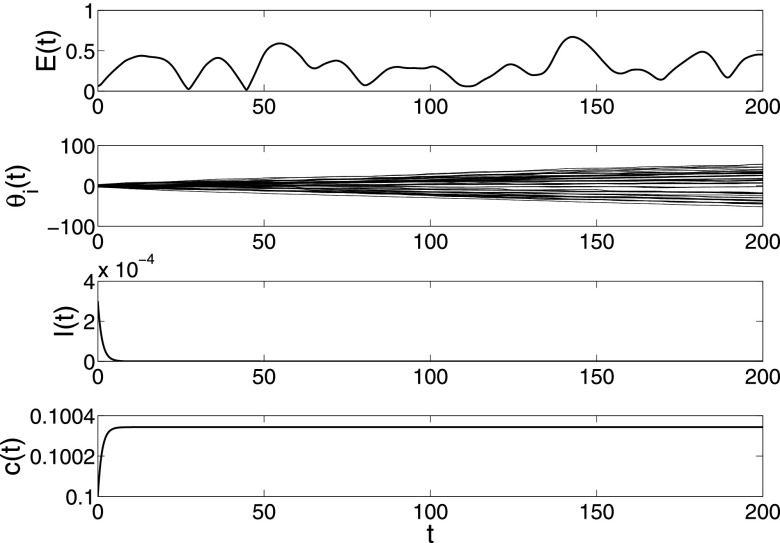
The changes of synchronization error *E*(*t*), phase $\theta_{i}$, epidemic prevalence *I*(*t*) and coupling strength *c*(*t*) in model (28) under parameters N=100,λ=0.05,α=0.8. Both the spreading network and dynamical behavior network have the same WS small-world network structure. There are only infected nodes with degree 3 and infection density $\rho_{3}=0.03$, and the other nodes are all susceptible in the beginning stage.

**FIG. 6.**
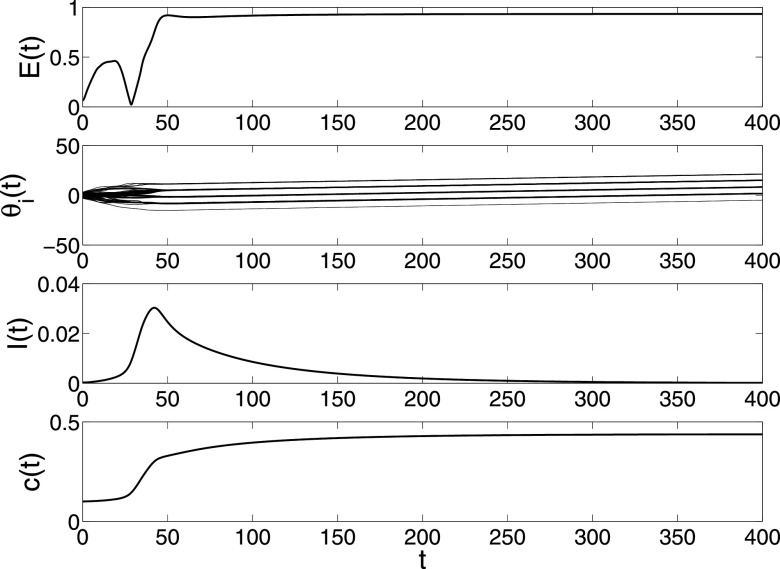
The changes of synchronization error *E*(*t*), phase $\theta_{i}$, epidemic prevalence *I*(*t*) and coupling strength *c*(*t*) in model (28) under parameters N=100,λ=0.2,α=0.8.

**FIG. 7.**
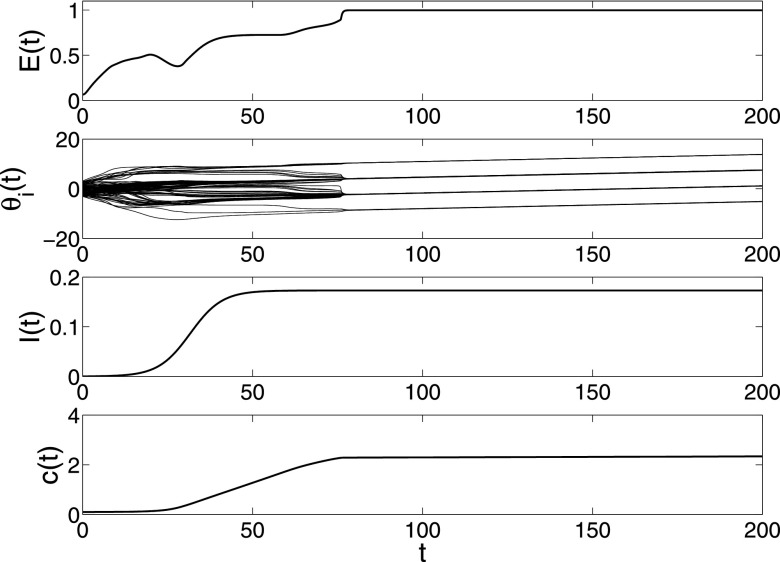
The changes of synchronization error *E*(*t*), phase $\theta_{i}$, epidemic prevalence *I*(*t*) and coupling strength *c*(*t*) in model (28) under parameters N=100,λ=0.2,α=1.

With the increasing spreading rates λ, we can see that the number of synchronous clusters in dynamical behavior network becomes smaller and the stable total density of spreading network becomes larger (see Figs. 5 and 6). Moreover, an interesting observation in Fig. 6 is that the total density fluctuates and goes to zero eventually. Then, by setting α = 1 we can see from Fig. 7 that the spreading behavior becomes endemic. From these simulations, we can conclude that the collective behavior of the dynamical behavior network can inhibit efficiently the spreading behavior. On the contrary, strong spreading behavior accelerates the collective behavior. These characteristics accord with many real dynamical networks very well.

## VI. CONTROL OF THE SPREADING NETWORK

This section will address the control problem of the spreading network (2) by adjusting its structure and awareness to collective behavior and then provide an effective control strategy to prevent or weaken the diffusion of the spreading behavior. The results show that the awareness is a critical factor for this control strategy, while the network structure seems relatively insignificant in this control process.

The change of network structure is performed by adjusting the rewiring probability *p* in WS small-world network. By increasing the probability *p* from 0 to 1, we can get a transition from a regular network to a random graph. The awareness can be adjusted by changing the parameter α in model (2), where smaller value of parameter α means greater awareness to collective behavior.

**FIG. 8.**
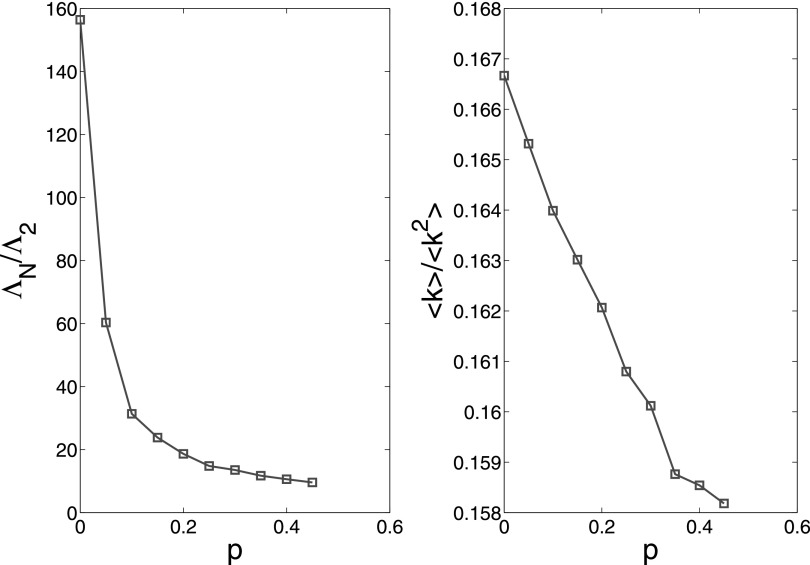
Both eigenvalue ratio $\Lambda_{N}/\Lambda_{2}$ and spread threshold $〈k〉/〈k^{2}〉$ decrease with increasing rewiring probability *p* in WS small-world network. This figure is obtained by 50 independent realizations.

As we known, the eigenvalue ratio $\Lambda_{N}/\Lambda_{2}$ of the adjacent matrix can quantify the synchronizability of the dynamical behavior network^17,18^ (the smaller this ratio is, the stronger synchronizability of the network), and the ratio $〈k〉/〈k^{2}〉$ denotes the spread threshold of the traditional SIS network model (i.e., the second equation in model (2) with ϕ(t)=1). By increasing the probability *p* in WS small-world network, we find the ratios $\Lambda_{N}/\Lambda_{2}$ and $〈k〉/〈k^{2}〉$ both decrease (see Fig. 8). This implies that the synchronizability is enhanced in the uncoupled dynamical behavior network, and the spread threshold decreases in the uncoupled spreading network. However, as these two networks are coupled by the form (2), we find that the change of network structure seems having relatively insignificant impact on the epidemics in this process, compared to the change of awareness.

*Initial condition setting:* The network size *N* = 100, α=0.5,σ=0.01,β=0.001. The initial infectious density $I_{d_{\min}}(0)=\frac{1}{Np(d_{\min})},\;I_{k}(0)=0$ for all $k\neq d_{\min}$ (i.e., just an individual with minimal degree $d_{\min}$ is infected). All simulations in this section are based on 50 independent realizations.

**FIG. 9.**
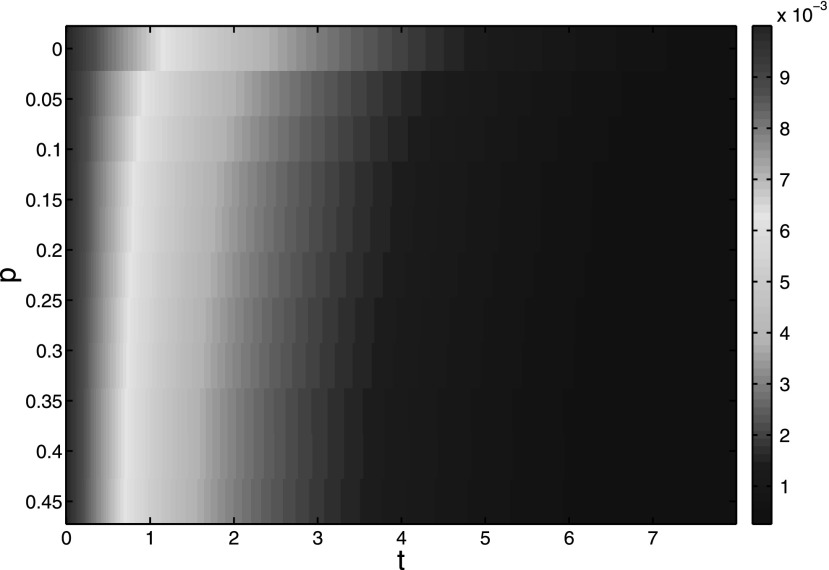
The epidemic prevalence of model (2) with disease-free equilibrium under λ = 0.2. The rewiring probability *p* increases form 0 to 0.45 with step length 0.05. The color point denotes the value of total infectious density *I*(*t*).

**FIG. 10.**
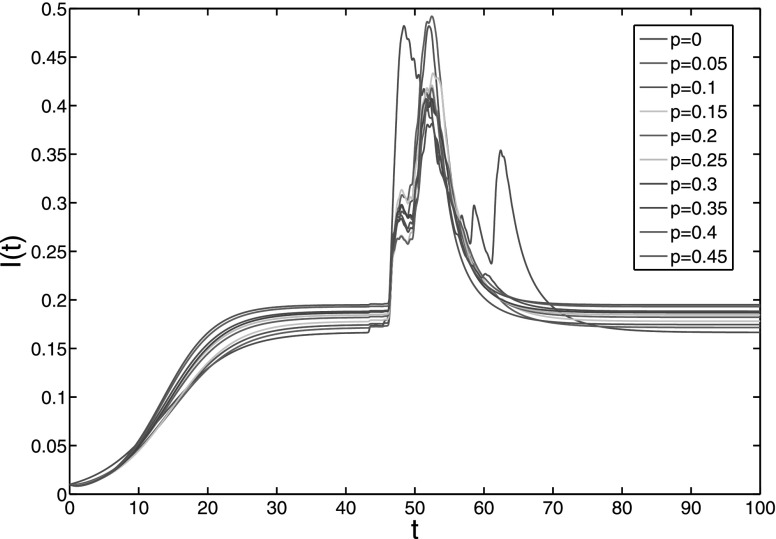
The epidemic prevalence of model (2) with endemic equilibrium under λ = 0.4. The rewiring probability *p* increases form 0 to 0.45 with step length 0.05.

**FIG. 11.**
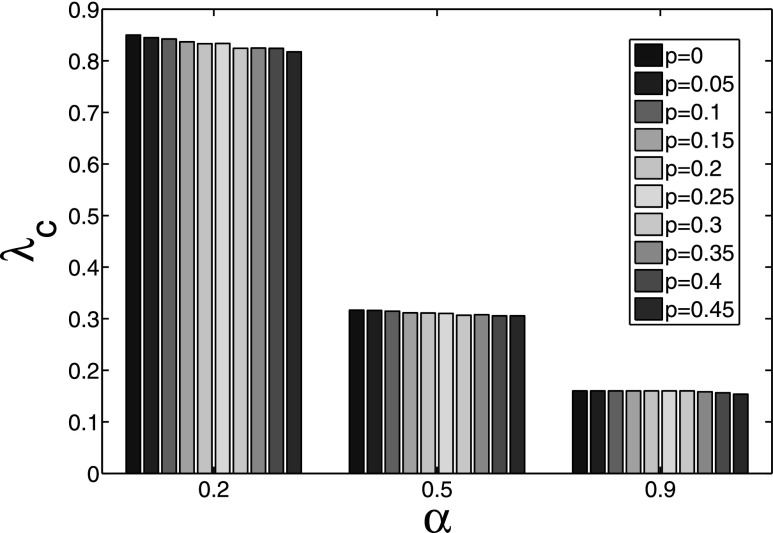
The spread threshold under different awareness degree α and rewiring probability *p*.

From Figs. 9 and 10, we can see that under fixed parameter α = 0.5, the change of network structure just plays trivial role in the spreading process, as the spreading prevalence *I*(*t*) does not vary obviously either in disease-free case or in endemic case (By performing simulations with more nodes, we have not found any essential difference to this simulation with 100 nodes. So, we can conclude the qualitative dependence of *I*(*t*) on *p* by this two figures). For other values of parameter α, the results are similar to this case. We can further observe this trivial influence in Fig. 11, where the spread threshold $\lambda_{c}$ seems fixed if the parameter α keeps constant. However, with the increasing of parameter α, the spread threshold $\lambda_{c}$ is decreased greatly. Therefore, the awareness is a critical factor for the spreading control strategy, and an effective control method is to enhance the awareness to collective behavior.

## VII. CONCLUSION

In summary, this paper has constructed several coupled models which can simulate collective and spreading behaviors on complex networks. Two concrete models are studied, respectively, where the spreading behavior is controlled by traditional SIS network model and the collective behavior is demonstrated by global synchronization and phase synchronization. The spread threshold of spreading network is obtained by using the stability theory method, and it depends on the network structure and individual awareness. The synchronization manifold of the dynamical behavior network is globally asymptotically stable if the spreading network can achieve an endemic state. Moreover, some numerical simulations are given to verify these theoretical results. We find that the collective behavior can inhibit spreading behavior, but, conversely, this spreading behavior can accelerate collective behavior. Finally, we study the impact of the change of network structure on spreading dynamics and find an effective control of spreading behavior is to enhance the awareness to collective behavior. This work may provide a basic framework to better understand and control such complex networks systems.
